# Making Blood: The Haematopoietic Niche throughout Ontogeny

**DOI:** 10.1155/2015/571893

**Published:** 2015-05-31

**Authors:** Mohammad A. Al-Drees, Jia Hao Yeo, Badwi B. Boumelhem, Veronica I. Antas, Kurt W. L. Brigden, Chanukya K. Colonne, Stuart T. Fraser

**Affiliations:** ^1^Discipline of Physiology, School of Medical Sciences, Bosch Institute, University of Sydney, Camperdown, NSW 2050, Australia; ^2^Laboratory of Bone Marrow and Stem Cell Processing, Department of Medical Oncology, Medical Oncology and Stem Cell Transplant Center, Al-Sabah Medical Area, Kuwait; ^3^Discipline of Anatomy & Histology, School of Medical Sciences, Bosch Institute, University of Sydney, Camperdown, NSW 2050, Australia

## Abstract

Approximately one-quarter of all cells in the adult human body are blood cells. The haematopoietic system is therefore massive in scale and requires exquisite regulation to be maintained under homeostatic conditions. It must also be able to respond when needed, such as during infection or following blood loss, to produce more blood cells. Supporting cells serve to maintain haematopoietic stem and progenitor cells during homeostatic and pathological conditions. This coalition of supportive cell types, organised in specific tissues, is termed the haematopoietic niche. Haematopoietic stem and progenitor cells are generated in a number of distinct locations during mammalian embryogenesis. These stem and progenitor cells migrate to a variety of anatomical locations through the conceptus until finally homing to the bone marrow shortly before birth. Under stress, extramedullary haematopoiesis can take place in regions that are typically lacking in blood-producing activity. Our aim in this review is to examine blood production throughout the embryo and adult, under normal and pathological conditions, to identify commonalities and distinctions between each niche. A clearer understanding of the mechanism underlying each haematopoietic niche can be applied to improving *ex vivo* cultures of haematopoietic stem cells and potentially lead to new directions for transplantation medicine.

## 1. Introduction

Haematopoietic stem and progenitor cells (HSPC) require signals from neighbouring cell types to maintain their self-renewing potential. The microenvironment that is responsible for maintaining this unique property of stem and progenitor cells is termed the niche. HSPC originate and expand in a number of very distinct niches in the mammalian conceptus. Shortly before birth, HSPC home to the bone marrow (BM) to reside there for the remainder of the mammal's life. The haematopoietic niche plays roles in supporting the initial production of HSPC, the expansion of HSPC to allow the embryo to survive, and the maintenance of HSPC in the BM maintaining homeostasis and may be activated in peripheral anatomical sites to respond to stress [1]. The role of the niche may therefore vary widely according to the developmental stage of the embryo or the stress the adult is placed under.

In contrast to embryonic stem cells and similar induced pluripotent stem cells, we are still unable to maintain HSPC indefinitely. Initial studies focused on stromal populations, often fibroblastic in nature, isolated from haematopoietic tissues such as the yolk sac, foetal liver, and BM. These stromal cells offered signals such as soluble factors and cell-cell interactions which supported the* ex vivo* or* in vitro* expansion of HSPC. Defining the mechanisms that niche cells orchestrate to maintain or expand HSPC under stress will improve the current therapeutic uses of blood stem and progenitor cells.

## 2. Blood Production or Haematopoiesis

No discussion of the haematopoietic niche can take place without discussing the haematopoietic cells themselves. However, we are focusing primarily on the niche rather than describing the blood lineages in detail. Numerous reviews specifically discuss different blood lineage production [2–5]. The haematopoietic system in the adult is responsible for the production of a broad range of different cell types from oxygen-transporting erythrocytes, the blood-clotting platelets, to the numerous forms of granulocytes through to the lymphoid branch with different T, B, NK, and innate lymphocytes. Dendritic cells, of various forms, as well as mast cells are also generated by the haematopoietic system. The haematopoietic system is therefore a complex array of different blood cell types performing a broad range of tasks to maintain homeostasis [5].

In the developing embryo, blood cells are the first cell type to become functionally mature highlighting the critical requirement for this lineage. A range of different blood cell types are also generated during embryogenesis which either are unique to the conceptus (e.g., primitive erythroid cells, foetal liver erythroid cells) or contribute to haematopoietic lineages with low turnover in the adult (microglia, Kupffer cells, and other tissue macrophages) [6, 7]. The first blood cells to appear have limited progenitor activity and it is not for several more days of embryonic development that cells with multilineage haematopoietic stem cell (HSC) activity arise. However, once adult-type (definitive) HSCs are generated, a clear hierarchy appears in which rare HSCs give rise to more frequent, lineage-committed progenitors. These progenitors in turn become more lineage-restricted, eventually giving rise to the massive numbers of mature blood cells needed. This hierarchy is critical in maintaining life in the adult mammal. An expansion of any stage or a blockade in differentiation can lead to pathological conditions ranging from leukaemia to anaemia.

In this review, we will discuss the cellular constituents of the microenvironments that help to establish, maintain, or reactivate this haematopoietic hierarchy from the first appearance of blood cells in the extraembryonic yolk sac through to the cells inhibiting blood production in the diseased and aged BM. We will primarily focus on the processes in the mouse as this is the best characterised model system for investigating mammalian haematopoiesis.

## 3. Prenatal Haematopoietic Niches

**Figure 1:**
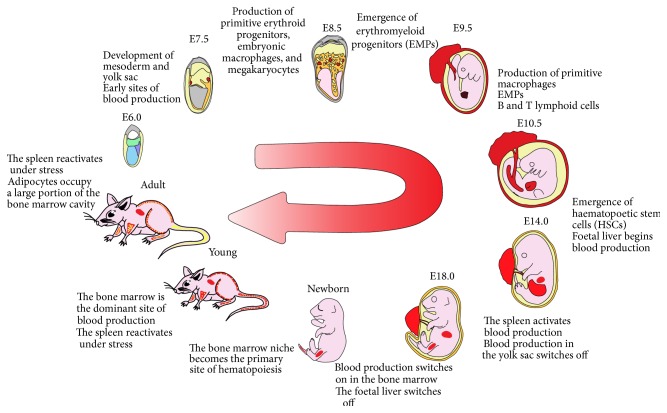
Timeline of haematopoiesis from early development to postnatal development in mice. This figure depicts the anatomical locations of haematopoiesis and the trafficking of haematopoietic stem cells (HSCs) and progenitor cells essential for maintaining haematopoiesis for life. Blood production begins at the mesoderm (blue, E6.0) and yolk sac (orange, all stages). From here, the first wave is initiated at the yolk sac blood islands (red, E7.0 and E8.5). Blood production is then shifted to the yolk sac and placenta (red, E9.5), with the latter providing blood to the foetus (pink) until birth. At E10.5, blood production initiates at the foetal liver and aorta-gonad-mesonephros (AGM) (red). The spleen initiates blood production at E14.0 (red) and continues to be a site of haematopoiesis after birth at time of stress. At E18.0, blood production shifts to the bone marrow (red), which remains the dominant site of haematopoiesis for life. After birth, adipocytes (yellow) begin to accumulate within the bone marrow and progressively increase as the mouse ages. Early development pictures modified from [17, 18].

The generation of blood cells during embryogenesis is complicated by the fact that an increasing number of anatomical sites have been proposed to give rise to blood.  Figure 1 shows the complex changes in anatomical location of blood production from the appearance of the first blood through to aged animals. For a thorough explanation therefore, we will be using the term conceptus, which includes the developing embryo or foetus as well as the placenta and the extraembryonic yolk sac. Mouse embryogenesis takes approximately 21 days from fertilisation to birth. Embryonic day of development will be abbreviated to E.

### 3.1. Initiation of Blood Production

**Figure 2:**
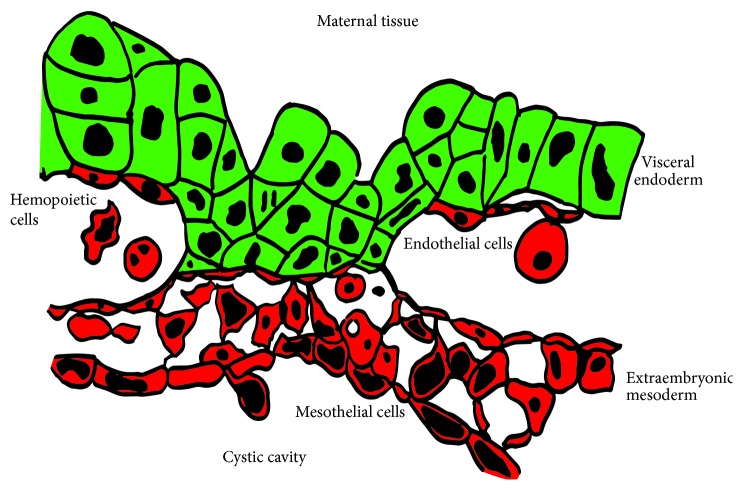
Histology of the yolk sac. The visceral endodermal cells form an epithelial sheet facing the maternal tissue (green). The mesoderm-derived tissue (red) includes vascular endothelial cells, haematopoietic cells, and a thin mesothelial layer facing the embryo proper.

The first site of blood production in mouse conceptus is the yolk sac (YS) which is a bilayered membrane that surrounds the developing embryo (see Figure 2). Haematopoietic activity occurs in the mesodermal layer of the vascularised visceral yolk sac (VYS), which contains blood vessels and haematopoietic cells. In addition to the extraembryonic mesoderm, the VYS is also composed of an outer epithelial layer of extraembryonic visceral endoderm (VE) made of columnar epithelial cells with a brush border [8]. The VE layer of the YS has a large absorptive surface and is involved in embryonic nutrition [9]. In addition to its role in transporting substances between the maternal and foetal environments, VE cells also contribute to haematopoiesis by secreting factors that induce commitment of mesodermal progenitors to the hematovascular fate [10, 11]. The developing endothelium and haematopoietic cells are in very close proximity to the visceral endodermal cells [8]. The VE itself is derived from an extraembryonic endodermal progenitor. The specification of VE identity is dependent upon the extraembryonic endodermal cells being exposed to bone morphogenetic protein-4 (Bmp4), leading to gene expression changes resulting in haematopoietic supportive activity [12]. Coculture of haematopoietic progenitors with YS-derived endodermal cell lines results in expansion of haematopoietic cells* in vitro* [13]. The transcription factor GATA4 is critical to VE formation [9]. Embryonic stem (ES) cells lacking GATA4 are unable to form blood islands demonstrating the role of the VE in haematopoietic cell induction [9]. Factors secreted by the VE can induce the primitive ectoderm (which is fated to become brain) to be respecified to become haematopoietic leading to epsilon (*ε*)-haemoglobin expression [10]. Indian Hedgehog (Ihh) produced by the VE activates expression of bone morphogenic protein-4 (Bmp4) in neighbouring mesodermal cells leading to haematopoietic initiation and patterning [10, 11]. Ihh also regulates vascular endothelial growth factor receptor 1 and 2 (VEGFR-1 and VEGFR-2) expression [14]. While both layers of the YS produce VEGF, mesodermal VEGF is unable to induce blood cell formation [15]. VEGF produced by the VE however is critical in blood and endothelial cell development in the YS demonstrating a clear role for the VE in the first haematopoietic niche [15, 16].

### 3.2. Primitive Haematopoiesis

The onset of YS haematopoiesis occurs from E7.5 in the mouse conceptus [19, 20]. This initial phase of blood production is termed primitive haematopoiesis [20–22]. During this phase, bands of primitive blood cells loosely associated with endothelial cells (the so-called blood islands) can be identified in the YS [23]. Primitive erythroid cells enter the circulation at approximately E9.0 [24]. Primitive erythroid progenitors (EryP-CFC) are maintained in the YS for close to 48 hours in close proximity to vascular endothelial cells and the VE described above. EryP-CFC express receptors for soluble growth factors such as c-Kit (receptor for stem cell factor, SCF), transforming growth factor-*β* (TGF*β*) receptor, erythropoietin receptor (EpoR), angiopoietin (Ang) receptors, and VEGFR-2 [20]. YS endothelial cells purified according to expression of VEGFR-2 express SCF, TGF*β*1, and Ang1 mRNA [20]. VE cells, isolated by virtue of GFP expression driven of the *α*-fetoprotein protein (AFP) promoter, express Ang1 mRNA [20]. VCAM-1^+^ mesenchymal cells in the YS have also been implicated as a niche cell controlling primitive erythroid cell maturation [25]. These cells have not been histologically identified as yet though it is likely that they represent the thin mesothelial layer of the YS [25]. VCAM-1 is a receptor for *α*4*β*1-integrin present on EryP-CFC at this stage of development in the YS [20, 26]. Collectively, primitive erythropoiesis is supported by signals from the neighbouring YS endothelial cells, signals diffusing from the visceral endoderm and possibly through direct cell-cell interactions with the mesothelium.

### 3.3. Definitive Haematopoietic Niches

The first wave of definitive (adult-type) haematopoiesis starts at E8.25 in the YS with erythromyeloid progenitors [22, 27]. Embryonic definitive erythrocytes are smaller than their primitive counterparts but larger than adult erythrocytes [28]. The second wave of definitive haematopoiesis is characterised by the production of definitive progenitor and stem cell populations including lymphoid progenitors and HSCs capable of long-term multilineage reconstitution of newborn and foetal recipients [29–31]. Despite the rapid changes in haematopoietic potential in this narrow developmental window, the impact of the other YS compartments is not well defined. It is currently unclear which signals from the YS niche are activating the definitive haematopoietic programme within the developing blood cells. However, we have recently observed that the secreted peptide gastrokine-2 is upregulated, transiently, in the YS VE cells close to the time at which haematopoietic activity diminishes in this tissue [32]. Gastrokine-2 protein was localised at the basal end of the VE cell facing the neighbouring endothelial cell, suggesting that this may be a signal from the VE influencing endothelial and possibly haematopoietic cell behaviour [32].

### 3.4. The Placental Haematopoietic Niche

*In vitro* culture studies showed that the embryonic component of mouse placenta forms a niche supporting haematopoietic progenitors [33]. HSPC are present in the mouse and human placenta [34–37]. Clusters of haematopoietic progenitor cells can be observed attached to placental vascular endothelial cells [38]. Similar to the early YS, endothelial cells in the placenta produce SCF whilst the haematopoietic progenitors expressed the SCF receptor c-Kit [38]. Three regions are clearly defined in the placenta: the outer maternal decidua; the middle spongiotrophoblast layer; and the vascularized labyrinth. Placental trophoblasts produce platelet-derived growth factor-*β* (PDGF*β*) to prevent the premature differentiation of HSPC, particularly erythroid progenitors [39]. The human placenta was identified as a niche supporting the terminal differentiation of primitive erythroid cells through interactions with macrophages [40].

## 4. Embryonic Haematopoietic Niches

### 4.1. Aorta-Gonad-Mesonephros (AGM)

The dorsal aorta, at the level of the developing gonadal and mesonephros (AGM), exhibits clusters of haematopoietic cells attached to the endothelial wall [21, 41, 42]. These haematopoietic clusters contain definitive HSC activity [43–45]. Explant studies confirmed that tissues located ventrally relative to the dorsal aorta induce HSPC activity [46]. In contrast, tissues located dorsal to the aorta lack HSPC supportive activity [46]. When liver or BM HSPC are cultured with AGM stromal cells, HSPC repopulating- and colony-forming potentials are preserved [47]. However, not all AGM endothelial populations have the same supportive abilities. Endothelial cells derived from the ventral region of the dorsal aorta support both HSC maintenance and differentiation. Meanwhile, cells from the urogenital subregion of the AGM support HSC maintenance but fail to induce HSC activity [47, 48]. Cytokines, soluble factors, and physical anchorage are part of the AGM microenvironment that play a role in supporting HSCs [48, 49]. Growth factors that enhance HSPC activity such as SCF, Flt3-ligand, interleukin-3 (IL-3), and Bmp4 are all expressed in the AGM [44, 50, 51]. Hedgehog signalling also plays a role in regulating HSC activity in the AGM [46]. Signalling from the nervous system also influences intraembryonic haematopoiesis [52]. Sympathetic nervous system mediators (catecholamines) regulate HSC emergence independent of blood flow [52]. Furthermore, catecholamine receptors are present on nascent HSCs, reinforcing the interplay between nervous system and haematopoiesis [52]. Other factors, such as retinoic acid signalling and blood shear stress, are also key regulators of embryonic blood production [53–55].

### 4.2. Foetal Liver Haematopoietic Niche

By E10.5–11.0, haematopoietic progenitors are migrating from the YS, AGM, and placenta to the foetal liver (FL). HSPC activity increases rapidly in this endoderm-derived tissue as the embryo matures. The FL haematopoietic niche consists of a variety of cell types including maturing haematopoietic cells themselves, sinusoidal endothelial cells, macrophages, stromal fibroblasts, and hepatoblasts (progenitors of hepatocytes). Hepatoblasts produce a broad range of haematopoietic growth factors including SCF, erythropoietin (Epo), thrombopoietin (TPO), and IL-6 which all support erythroid cell development [56]. The role of hepatoblasts in supporting haematopoiesis is best exemplified by mice lacking the tyrosine kinase Map2k4. These mice fail to form hepatoblasts, showing a significant reduction in Epo and SCF expression and a concomitant impairment in blood cell production, in particular erythropoiesis. HSPC express *β*1-integrin which interacts with vitronectin and fibronectin in the extracellular matrix produced by hepatoblasts [57]. This deposition of extracellular matrix by hepatoblasts is regulated by autocrine production of TGF*β* [57]. Hepatoblasts also produce IL-7 to support lymphoid cell development [58]. A series of reports detailed the surprising use of an anti-CD3e antibody to purify a FL population which supported HSC maintenance. This population expresses a range of soluble factors including insulin-like growth factor-2 (IGF-2) and angiopoietin-like proteins 2 and 3 (Angptl2 and Angptl3). These cells have since been shown to express the hepatoblast markers albumin and Dlk, GFP-driven by the AFP promoter, and surface SCF. These cells also produce the chemokine CXCL12, also known as stromal-derived factor (SDF) [59]. Collectively, this data suggests these cells are likely to be hepatoblastic in origin. The FL niche was modeled* in vitro* following the generation of AFT024, a mouse FL stromal cell line. This cell line is highly supportive of HSPC and has been characterised at the transcriptome level to identify regulators of HSPC activity [60].

HSPC, expressing the surface marker Endothelial Protein C Receptor (EPCR), interact with sinusoidal endothelial cells [61]. These HSPC were observed in both the luminal and parenchymal aspects of the Lyve-1^+^ sinusoidal endothelial cells extracellular matrix made up of laminin and fibronectin [61]. Very recent findings in the zebrafish show that the arrival of blood progenitors in the perivascular niche leads to profound changes in the niche itself. Endothelial cells actively surround HSCs once they have entered the perivascular space [62]. A similar process was observed in the mouse FL. When transgenic labelled HSPC entered the FL parenchyma, vascular endothelial cells congregated around the HSPC. The HSPC then anchor to perivascular cells and orient their mitotic division planes according to the perivascular cell body they have adhered to [62]. These intriguing findings show that the niche is responsive to the behaviour of HSPC.

FL macrophages form a supportive microenvironment termed the erythroblastic island (EBI). Although the role of EBI is better defined in the BM (will be described later), FL EBI macrophages support the terminal differentiation of primitive erythroid cells [63, 64]. This interaction is dependent upon *α*4*β*1-integrin present on the primitive erythroid cells binding to VCAM-1 expressed by the FL EBI macrophage [63]. The precise role is unclear; however one important element is the engulfment and destruction of millions of expelled erythroid nuclei. FL macrophages lacking DNAse II, the primary DNA-degrading enzyme, show a massive uptake of erythroid nuclei which they are unable to digest. This leads to the macrophage rupturing releasing inflammatory mediators and killing the embryo [65].

## 5. The Bone Marrow Haematopoietic Niche

The bone marrow (BM) is the predominant blood-producing site of adult mammals. The migration of the haematopoietic activity from the embryonic and foetal tissues, described above, to the foetal BM is only now being defined at the molecular level. Investigating foetal BM niche formation will help us to define the essential elements of the niche as it maintains blood production in the newborn, juvenile, and adult mammals.

### 5.1. Foetal Bone Marrow Haematopoietic Niche Formation

The long bones of the mouse foetus begin to develop at approximately E14.5 and arise from mesodermal progenitors. At E15.5, the mouse long bones are cartilaginous bone templates lacking blood vessels. One day later in development, vessels can be detected in the periosteum (the layer surrounding the developing bone) and the epiphyseal plates found at each end of the long bones [66]. Calcification and vascularisation of BM cavity then begin, primarily in the middle region of the long bone. At this stage, collagen type 1, alpha 1- (Col1*α*1-) expressing osteoblasts emerge in the periosteum [66]. The middle cavity region is dominated by CD31^+^ endothelial cells forming blood vessels and c-Kit^+^ Sca-1^+^Lineage^neg^ (KSL) HSC can be detected. By E17.5, Col1*α*1^+^ osteoblasts occupy the middle BM cavity and expand alongside CD31 expressing vascular endothelial cells, both proliferating toward the epiphyseal plate [66]. The BM will eventually fill the medullary cavities of bones throughout the skeleton. The BM cavity can be subdivided into four regions: endosteal, subendosteal, central, and perisinusoidal regions. The endosteum is tissue lining the bone surface and facing the marrow cavity or the trabeculae of the spongy bone within the cavity and is mostly composed of osteoblasts though osteoclasts are also important components of this microenvironment [67]. Mice lacking the transcription factor Osterix (*Osx*) fail to form mature osteoblasts and mineralised bone matrix [68].* Osx* ^−/−^ foetal BM is vascularised normally but lacks functional HSPC [66].* Osx* ^−/−^ mice are therefore a useful model for assessing the role of osteoblasts in establishing the foetal BM niche.

Homing, adhesion, and retention of HSPC in the haematopoietic BM niche and their migration to corresponding microenvironments are controlled by chemotactic factors. Inactivation of the genes encoding the chemokine CXCL12 or its receptor CXCR4 led to significant decrease of HSC frequency in E17.5 foetal BM [69]. Foetal liver HSC frequency was not affected by* Cxcr4*-deficiency [69].* Cxcr4*-deficient foetal BM is hypocellular with a severe reduction in myeloid cell frequency [70]. Mice lacking the genes encoding serum response factor (*Srf*) or myocardin-related transcription factor (*Mrtf*), which is regulated by Srf, also show normal FL HSPC frequency and exhibit a failure of HSPC to migrate to the foetal BM [71].* Srf*-deficient HSPC fail to respond to the chemotactic cues of CXCL12. This foetal BM colonisation defect is phenocopied in mice lacking either* Mrtfa* or* Mrtfb* which show defective cell motility and actin remodelling [71]. CXCL12 and* Scf* gene expression was upregulated in* Osx* ^−/−^ mutant E17.5 BM [66]. Collectively, these findings suggest a BM-specific mechanism of niche formation dependent upon the CXCL12-CXCR4 axis and associated molecules.

### 5.2. The Neonatal and Juvenile Bone Marrow Haematopoietic Niche

Profound physiological changes take place at birth. The newborn must breathe and digest food for the first time; the connection to the maternal circulation via the placenta and YS has ceased and, perhaps most importantly, the newborn is exposed to the external environment resulting in the activation of both the innate and adaptive immune systems. Haematopoiesis needs to accommodate these changes in oxygen transport and immune response. Surprisingly, very little is known about the immediate changes in the neonatal niche in spite of the profound shift in physiological activity. However, calcium ions originating from the bone matrix are clearly important. Mice lacking the calcium receptor (CaR) have hypocellular marrow just after birth [72].* CaR* ^−/−^ HSPC fail to migrate to the endosteal niche as they cannot detect the increase in free calcium ions released from the bone surface [72].* CaR* ^−/−^ HSPC show defects in binding to the extracellular matrix proteins fibronectin and collagen type 1 [72]. As the juvenile mouse matures, the bone matrix becomes more heavily ossified (mineralised). This complex process is critical in establishing a stable haematopoietic niche and relies on a broad range of regulators from transcription factors such as Runx2, phosphoproteins such as bone sialoprotein (Bsp), and osteocyte proteins such as Saa3. Deletion of genes encoding these proteins leads to abnormal mineralisation of the bone matrix potentially compromising the BM.

## 6. The Adult Bone Marrow Haematopoietic Niches

**Figure 3:**
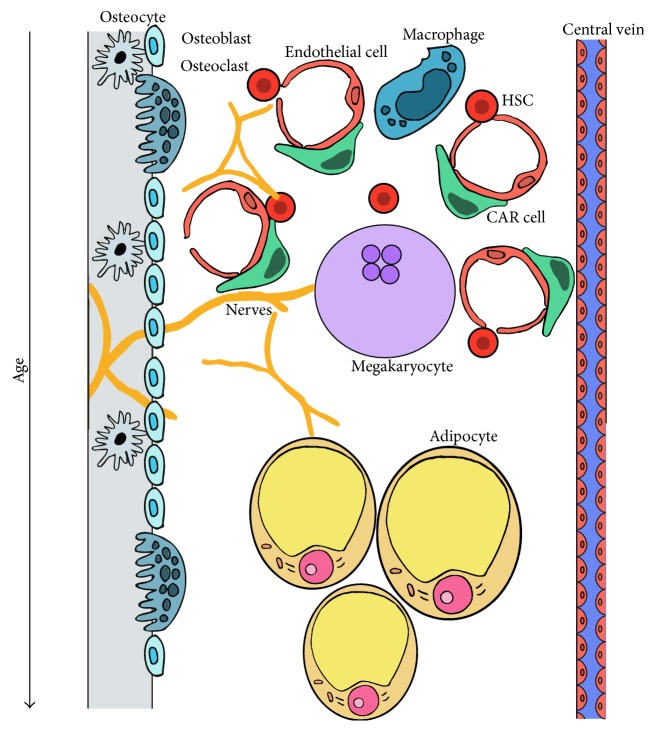
Cellular interactions within the adult bone marrow niche. The bone marrow niche is home to a variety of cell types that promote or inhibit the maintenance of HSCs and haematopoietic progenitors. The endosteal niche and perivascular niche can be clearly discerned. Adipocytes fill the marrow with age, inhibiting blood cell production.

The BM serves as the main HSPC niche for adult eutherian mammals though the bones do not appear to vary in their HSC frequency [73]. The concept that there are specific cell types that can orchestrate a BM niche was strengthened by the discovery of a transplantable CD146-expressing cell in the human BM which, when transplanted into mice, could give rise to an entirely new BM niche which could support mouse HSC activity [74]. HSC can be found in most regions of the long bone; the trabeculated regions of the metaphysis are the preferred site of homing compared to the epiphysis (the endplates) or the diaphysis (the shaft of the long bone) [75, 76]. However, it is now clear that there are at least two anatomically distinct niches: the endosteal niche close to the bone surface and the perivascular niche associated with arterioles.  Figure 3 simplifies the complex cellular compartments in the adult BM nice.

### 6.1. The Endosteal Niche of the Adult Bone Marrow

High-resolution cytometric analysis of the adult BM has shown that HSCs preferentially localise to two distinct niches, the perivascular niche and the endosteal niche [77]. Mouse and human osteoblasts support HSPC [78]. The endosteal niche exhibits unique physiological properties including hypoxia and a greater concentration of free calcium ions coming from the bone surface [72, 77]. HSCs adhere to the osteoblasts [77, 79, 80]. Mice expressing a constitutively active form of parathyroid hormone led to an expansion in osteoblast number and HSC frequency [81]. Mice with conditional deletion of BMPR1a possess increased numbers of N-cadherin^+^ osteoblastic cells. These in turn interact directly with HSC. This leads to an increased HSC frequency [82]. Conversely, transgenic ablation of osteoblasts led to a severe reduction in HSC frequency [83]. Inhibition of osteoclast function has been associated with reduced HSPC frequency [84]. However, osteopetrotic mice, which lack functional osteoclasts, show overgrowth of mineralised bone matrix and increased levels of HSPC [85]. This report challenged previous reports suggesting that osteoclasts may play a role in the BM haematopoietic niche.

Cooperative regulation among cytokine signals and cell adhesion molecules is required for HSCs maintenance or activation. Many factors including cytokines, chemokines, adhesion molecules, and transcription factors regulate the HSC quiescence in the endosteal niche. Some of the molecules defined in this process include Tie2 and angiopoietin-1 [86]; SCF and its receptor c-Kit [87]; CXCL12 [88]; thrombopoietin [89], Jagged1 interactions with Notch receptors [81]; osteopontin [90]; and N-cadherin which became a lightning rod of controversy in the field. N-cadherin expression was reported in the endosteal HSC niche [81, 82, 86] though its role remained unclear. However, N-cadherin is not required for HSCs maintenance though it may play a role in osteoblastic homophilic interactions [91, 92].

### 6.2. The Perivascular Niche

A niche supporting HSC activity has been identified in close proximity to the blood vessels of the adult BM and has been termed the perivascular niche [79]. HSCs interact with a perivascular mesenchymal cell type which has become the focus of many research groups. Imaging of the adult mouse BM revealed that the majority of quiescent HSCs are situated close to arterioles [93]. Cells expressing GFP under the control of the Nestin promoter (Nes-GFP) localise in the perivascular region. Although they constitute only a small fraction of BM cells, once purified these cells were shown to contain essentially all of the mesenchymal stem cell (MSC) activity of the marrow [94]. Even more striking, these Nes-GFP^+^ MSC were found to sit adjacent to HSC. Nes-GFP^+^ MSC produce soluble factors that support HSC maintenance such as CXCL12 and SCF. This led to the proposal that MSC form a niche with HSC directly and maintain HSC activity [94].

Two distinct populations could be detected according to GFP transgene expression in the BM. Cells expressing high levels of the Nestin-GFP transgene (Nes-GFP^bright^) localise to the perivascular region, exhibit pericyte-like morphology, and express the pericyte marker NG2 and *α*-smooth muscle actin. These are found exclusively around arterioles [93]. Dormant HSCs are found in close proximity to these Nes-GFP^bright^ cells. Genetic ablation of the NG2-expressing Nes-GFP^bright^ population resulted in HSC migrating away from the arterioles. In contrast, the more abundant cells expressing low levels of the Nestin-GFP transgene (Nes-GFP^dim^ cells) are reticular in shape and associate with sinusoids [93].

Recently, much attention has been given to the role of the sympathetic nervous system forming the adult BM niche. Circadian noradrenalin secretion by the sympathetic nerves negatively regulates CXCL12 expression in the BM. This in turn reduces mobilisation of HSC from the BM niche [95]. Denervation of sympathetic nerves resulted in loss of HSC; however other BM components such as endothelial cells, MSC, and osteoblasts remained intact [96]. These sympathetic nerves were also found to be ensheathed by autonomous, nonmyelinating Schwann cells which are in direct contact with HSCs and lie parallel to blood vessels [96]. These glial cells regulate activation of TGF*β* and HSC quiescence via TGF*β*/Smad signalling [96]. The sympathetic nerve regulation of HSC maintenance is mediated indirectly by Nestin^+^ MSC expressing the *β*3-adrenergic receptor [94]. Both adrenergic receptors *β*3 and *β*2 were reported to regulate HSC mobilisation [94].

Similar haematopoietic defects were seen in transgenic mice with neurological abnormalities. UDP-galactose ceramide galactosyltransferase (*CGT*) is required for the synthesis of galactocerebroside and sulfatide required for myelin [97].* CGT* ^−/−^ mice exhibit altered fibronectin network in the BM and a pronounced reduction in CD45^−^ VCAM-1^+^ stromal cells numbers [97]. Diminished HSC mobilisation from the BM was also observed in these* CGT* ^−/−^ mice after following granulocyte colony-stimulating factor (G-CSF) or fucoidan administration [98].

The molecular mechanisms regulating this niche are becoming more clearly defined. SCF is clearly a critical factor in regulating the adult BM niche. Ding and colleagues performed an exhaustive analysis of the cell types expressing SCF in the marrow using the *Scf* ^*gfp*/+^ mouse [99]. Endothelial cells and perivascular cells express GFP in this transgenic model. Conditional deletion of* Scf* in osteoblasts or Nestin-expressing cells had no effect on HSC frequency. However, specific loss of* Scf* in endothelial cells expressing Tie2-Cre led to significant loss of HSC frequency. The role of perivascular cell-derived SCF was confirmed by conditional deletion in leptin-receptor (LepR)-Cre-expressing cells. Intriguingly, this led to a reduction in BM HSC frequency with a concomitant increase in splenic HSC numbers. No change was observed during developmental stages demonstrating that SCF originating from the perivascular mesenchymal cells regulates the BM niche specifically. A similar phenotype was observed in vitamin D receptor (*VDR*) null mutant mice which show reduced BM HSC frequency and enhanced splenic HSC numbers [100].

CXCL12 is crucial to HSC homing to the BM. A population of CXCL12-abundant reticular (CAR) cells was observed in the vascular niche [88, 101–103]. Conditional deletion of CXCR4 results in profound reduction in HSC frequency [88]. Conditional deletion of CXCL12 from* Osx*-expressing BM CAR cells and osteoblasts resulted in HPC mobilization and decline in B lymphoid progenitors [102]. Therefore, signalling between CXCL12 from CAR cells and CXCR4 on HSC maintains HSC self-renewal, proliferation, and migration. CAR cells are bipotent and are able to give rise to both osteoblasts and adipocytes as will be discussed later in this review [103].

### 6.3. Other Cells of the Marrow Which Support Haematopoiesis

HSCs reside in close proximity to megakaryocytes [104, 105]. Ablation of megakaryocytes reduces HSC proliferation and engraftment [105, 106]. Thrombopoietin (TPO) administration of megakaryocyte-ablated mice restores HSC function [105]. Cxcl4, which megakaryocytes produce, inhibits HSC proliferation, reduces HSC numbers, and decreases engraftment [104]. An increase in HSC number, proliferation, and repopulating activity was observed in* Cxcl4*-deficient mice [104]. Megakaryocytes also maintain HSC quiescence through TGF*β* signalling under homeostatic conditions and promote HSC expansion via FGF-1 production under stress conditions [107]. High-resolution imaging* in vivo* revealed the colocalisation of HSCs with FoxP3^+^ regulatory T (Treg) cells on the endosteal surface [108]. However, whether this interaction is biologically relevant in homeostatic HSPC maintenance is unclear.

## 7. Haematopoietic Progenitor Niches

The expansion and maturation of committed haematopoietic progenitors require specific microenvironments. Here, we describe several critical niches regulating haematopoietic progenitor development into functionally mature cells.

### 7.1. Erythroblastic Islands Are Essential Niches for Erythropoiesis

**Figure 4:**
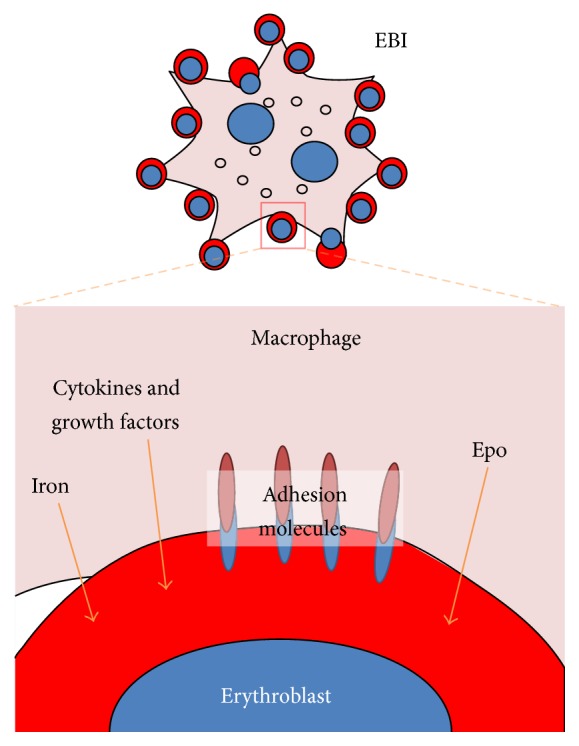
The erythroblastic island provides a niche for erythroid development. Central macrophage anchors erythroblasts in the erythroid niche via adhesion molecules. Within the EBI, the central macrophage supports erythropoiesis in several ways, such as providing iron for haemoglobin synthesis, cytokine, and growth factors as well as engulfing expelled erythroid nuclei.

Erythroblastic islands (EBI) are a specialised niche found where mammalian erythroblasts proliferate and differentiate and are described in Figure 4. Discovered by Marcel Bessis in the 1950s, EBI were first described as multicellular structures with developing erythroblasts at various stages of differentiation surrounding a central macrophage [109–111]. EBI have also been isolated from the foetal liver [63, 112] and spleen [113, 114]. The clinical significance of this niche was demonstrated in animal models of human haemoglobinopathies [115, 116]. The central macrophage of the EBI was proposed to be a “nurse” cell for erythroid development with macrophages providing iron to developing erythroblasts for heme synthesis [109]. However, there is still no direct evidence that the central macrophage is providing iron to the surrounding erythroblasts. Cytokines such as RCAS [117], TRAIL [118], and IGF-2 [119] are produced by EBI central macrophages. Another proposed function of the EBI central macrophage is to engulf and destroy extruded erythroid nuclei. Phosphatidylserine (PS) is exposed on the outer leaflet of the plasma membrane of the extruded erythroid nuclei [120]. EBI macrophages actively phagocytose extruded erythroid nuclei via the PS receptor, Tim-4 [63, 121]. After phagocytosis, the macrophages degrade the nuclei. DNaseII and its regulator, erythroid Kruppel-like factor-1 (Klf1), are essential in this process [65, 120, 122]. Embryos with targeted deletions of retinoblastoma tumour suppressor (Rb) protein,* c-Maf*, or the cytoskeletal protein paladin die* in utero* with defects in EBI function [112, 123, 124].

### 7.2. B Lymphoid Niche

B lymphoid progenitors reside in close proximity to the endosteum and migrate towards the central blood vessels in the BM as they mature [125]. VCAM-1^+^ CXCL12^+^ expressing reticular cells were among the first B-cell-specific niche to be identified [101]. These cells lack endothelial and endosteal markers and are distant from the endosteal surface. Other cell types thought to play a role include CXCL-12-expressing osteoblasts and IL-7-expressing cells [83]. B-cell progenitors attach to CXCL-12-expressing cells and then relocate and attach to IL-7-expressing cells as they mature into pro-B-cells progenitors [101]. After naive B-cells are sensitized by antigens in the peripheral lymphoid organs, resultant plasma cells migrate to the marrow which then serves as a specific niche for these mature cells [101]. This niche offers prerequisite soluble factors (Blimp-1, CXCL12, APRIL, IL-5, IL-6, and TNF-*α*) as well as necessary cell-cell contacts through CD44 and CD28. In the absence of IgE^+^ DX5^+^ basophils, isolated plasma cells rapidly die. Addition of basophils enabled plasma cells survival [126]. F4/80^+^ Gr-1^low^ Siglec-F^+^ eosinophils associate with plasma cells and supply IL-6 and APRIL for plasma cell survival [127]. Depletion of eosinophils in mice results in apoptosis of plasma cells [127]. Plasma cells also interact with megakaryocytes, which secrete APRIL and IL-6. Mice deficient in the thrombopoietin receptor, c-Mpl, have impaired megakaryopoiesis and show reduced numbers of plasma cells [128].

## 8. Normal versus Stressed Splenic Microenvironments

Red blood cells, or erythrocytes, are normally generated from erythrocyte progenitor cells residing inside the BM in a process termed medullary erythropoiesis. The erythrocyte production rate increases dramatically during anaemia [129]. To recover from anaemic stress, erythroid progenitors migrate from the BM to extramedullary sites such as the spleen, which act as secondary sites for erythrocyte production. This process is termed stress erythropoiesis and is a critical phase in the recovery from haemorrhage or diseases causing anaemia [130]. Stress erythropoiesis often manifests as a dramatic increase in spleen size (termed splenomegaly) and is most notable in chronic haemolytic conditions such as thalassaemia and sickle cell anaemia but is also observed in many haematological diseases such as myelofibrosis, leukaemia, and lymphoma [129]. Bmp4 and Hedgehog signalling act together with Epo, SCF, and hypoxia to encourage extramedullary haematopoiesis in the spleen during stress [131, 132]. Hedgehog signalling is also required for the recovery from anaemia [133]. The splenic stroma consists of vascular and lymphatic endothelial cells, marginal zone macrophages, follicular dendritic cells, fibroblastic reticular cells, marginal reticular cells, and red pulp fibroblasts. The erythroblastic island is the only known splenic stromal cell which has been examined during anaemic stress. Forssman antigen^+^ F4/80^+^ macrophages extend their cytoplasmic processes around erythroblasts in the spleen after irradiation and transplantation [113]. MSS31 endothelial-like cell lines isolated from newborn mouse spleen selectively supported the maturation and enucleation of the erythroid progenitor cells by direct cell-cell contact [134]. Fibroblast-like SPY3-2 cell line expresses high levels of SCF and low levels of granulocyte macrophage colony-stimulating factor and IL-3 and can support erythroid differentiation* in vitro* [135].

## 9. Inhibition of the Haematopoietic Niches

As mammals age, fatty marrow predominantly takes over the BM compartment [136]. BM adipocytes, or yellow adipose tissue (YAT), are often dismissed as simple “space-fillers” that lay dormant in the marrow [137]. YAT may also serve as an emergency energy reservoir [138]. However, it has been recently shown that YAT plays an active role within the haematopoietic microenvironment influencing haematopoiesis and osteogenesis [139]. YAT originates from BM MSC, the same precursors that give rise to osteoblasts and haematopoietic cell types. YAT responds to systemic changes in energy metabolism. This is most evident in ageing where there are large changes in YAT volume. As ageing individuals show increased BM YAT and anaemia, it was proposed that BM adipocytes may inhibit blood production. By comparing the haematopoietic recovery of wild-type and fatless A-ZIP/F1 mice after lethal irradiation, it was observed that the lack of adipogenesis in the fatless mice enhanced haematopoietic recovery [139]. This was due to the enhanced engraftment of short-term progenitors in the BM compartment. Ablation of YAT also improved osteogenesis [139].* In vitro* cultures of BM-derived adipocytes yielded a reduced expansion of haematopoietic cells, indicating that adipocytes release diffusible inhibitors of haematopoiesis. Secretion of neuropilin-1, lipocalin 2, adiponectin, and TNF-*α* from adipose tissue can inhibit proliferation of haematopoietic cells [139]. However, adiponectin can support HSC proliferation [140]. Transgenic ablation of CAR cells revealed their essential role in the niche and demonstrated the potential of these cells to form both osteoblasts and adipocytes [103]. The transcriptional regulator Foxc1 is highly expressed by CAR cells. Conditional deletion of* Foxc1* led to a profound change in CAR cell fate. Rather than giving rise to both osteoblasts and adipocytes, a lineage bias was observed with a far greater number of mature adipocytes forming in the BM [141]. This in turn led to a pronounced decrease in HSPC activity. CAR cells also expressed the adipogenesis markers leptin receptor and peroxisome proliferator-activated-receptor-*γ*. Enforced expression of Foxc1 in the preadipocytic cell stromal cell line OP9 led to enhanced haematopoietic supporting activity and loss of adipogenic activity [141]. Collectively, these findings demonstrate that Foxc1 is acting as a rheostat within critical niche cells, specifying whether the marrow will support or inhibit haematopoiesis.

## 10. Commonalities and Distinctions between the Different Haematopoietic Niches

Assessing the cellular and signalling components of all of the critical haematopoietic niches throughout ontogeny was a revealing exercise. Commonalities between these profoundly distinct niches become obvious. Not surprisingly, SCF and its receptor c-Kit were observed to play critical roles in extraembryonic, embryonic, foetal, and adult blood production as well as during recovery from stress. SCF and Hedgehog signalling are significant players in haematopoiesis ranging from the first primitive erythroid cells through to extramedullary haematopoiesis in the spleen during anaemia. Likewise, Bmp4 signalling was highlighted as being important in numerous niches including the YS, AGM, BM, and anaemic spleen. Hypoxia is also clearly important in a number of niches. The role of blood flow is critical in the AGM for HSPC development. It may also play a role in shutting down primitive erythroid progenitor activity in the YS. Similar rheological signals are less likely to be important in the parenchyma of the FL or BM. The distinction between foetal and perinatal niches was highlighted by the importance of CXCL12 and its receptor CXCR4 in the BM. This signalling system plays a far less significant role in the early embryonic stages of haematopoiesis though it is critical in numerous elements of adult haematopoietic production.

## 11. Application of Our Understanding of the Haematopoietic Niche

How can we apply our understanding of the different haematopoietic niches to medical technology and practice? The deconstructionist approach has been flipped recently with the development of “bone marrow-on-a-chip.” Microfluidic cultures of marrow maintained HSPC* in vitro* for at least 1 week demonstrating that the critical microenvironment for controlling HSC activity can be engineered [142]. This could improve current models for drug development and screening. Animal experimentation could be reduced or replaced as human “bone marrow-on-a-chip” systems could be used to assess the toxicity or efficacy of novel compounds on the human haematopoietic system. This system can be scaled up allowing for high throughput screening of new therapeutical compounds.

Perhaps the most medically relevant utility of the niche is as a system for the* ex vivo* expansion of HSPC for transplantation. Currently, we cannot maintain HSPC indefinitely* in vitro* or* ex vivo* in the same way we can maintain embryonic stem or induced pluripotent stem cells. One of the “holy grails” of haematology is therefore identifying the factor or combination of factors that can maintain HSPC indefinitely in an* ex vivo* setting. This would have massive repercussions for transplantation and transfusion medicine, as it could allow for the correction of genetic lesions in HSPC, thus allowing for effective engraftment after transplantation. This could be applied to a broad range of haematological disorders affecting millions of people such as sickle cell anaemia, *β*-thalassaemia, immunodeficiencies, and blood-clotting disorders.

## References

[1] Wang L. D., Wagers A. J. (2011). Dynamic niches in the origination and differentiation of haematopoietic stem cells. *Nature Reviews Molecular Cell Biology*.

[2] Orkin S. H., Zon L. I. (2008). SnapShot: hematopoiesis. *Cell*.

[3] Fraser S. T. (2013). The modern primitives: applying new technological approaches to explore the biology of the earliest red blood cells. *ISRN Hematology*.

[4] Baron M. H., Isern J., Fraser S. T. (2012). The embryonic origins of erythropoiesis in mammals. *Blood*.

[5] Rieger M. A., Schroeder T. (2012). Hematopoiesis. *Cold Spring Harbor Perspectives in Biology*.

[6] Ginhoux F., Greter M., Leboeuf M. (2010). Fate mapping analysis reveals that adult microglia derive from primitive macrophages. *Science*.

[7] Perdiguero E. G., Klapproth K., Schulz C. (2015). Tissue-resident macrophages originate from yolk-sac-derived erythro-myeloid progenitors. *Nature*.

[8] Haar J. L., Ackerman G. A. (1971). Ultrastructural changes in mouse yolk sac associated with the initiation of vitelline circulation. *Anatomical Record*.

[9] Bielinska M., Narita N., Heikinheimo M., Porter S. B., Wilson D. B. (1996). Erythropoiesis and vasculogenesis in embryoid bodies lacking visceral yolk sac endoderm. *Blood*.

[10] Belaoussoff M., Farrington S. M., Baron M. H. (1998). Hematopoietic induction and respecification of A-P identity by visceral endoderm signaling in the mouse embryo. *Development*.

[11] Dyer M. A., Farrington S. M., Mohn D., Munday J. R., Baron M. H. (2001). Indian hedgehog activates hematopoiesis and vasculogenesis and can respecify prospective neurectodermal cell fate in the mouse embryo. *Development*.

[12] Artus J., Douvaras P., Piliszek A., Isern J., Baron M. H., Hadjantonakis A.-K. (2012). BMP4 signaling directs primitive endoderm-derived XEN cells to an extraembryonic visceral endoderm identity. *Developmental Biology*.

[13] Yoder M. C., Papaioannou V. E., Breitfeld P. P., Williams D. A. (1994). Murine yolk sac endoderm- and mesoderm-derived cell lines support in vitro growth and differentiation of hematopoietic cells. *Blood*.

[14] Byrd N., Becker S., Maye P. (2002). Hedgehog is required for murine yolk sac angiogenesis. *Development*.

[15] Damert A., Miquerol L., Gertsentein M., Risau W., Nagy A. (2002). Insufficient VEGFA activity in yolk sac endoderm compromises haematopoietic and endothelial differentiation. *Development*.

[16] Miquerol L., Gertsenstein M., Harpal K., Rossant J., Nagy A. (1999). Multiple developmental roles of VEGF suggested by a LacZ-tagged allele. *Developmental Biology*.

[17] Yoder M. C. (2014). Inducing definitive hematopoiesis in a dish. *Nature Biotechnology*.

[18] Ginhoux F., Lim S., Hoeffel G., Low D., Huber T. (2013). Origin and differentiation of microglia. *Frontiers in Cellular Neuroscience*.

[19] Palis J., Robertson S., Kennedy M., Wall C., Keller G. (1999). Development of erythroid and myeloid progenitors in the yolk sac and embryo proper of the mouse. *Development*.

[20] Isern J., He Z., Fraser S. T. (2011). Single-lineage transcriptome analysis reveals key regulatory pathways in primitive erythroid progenitors in the mouse embryo. *Blood*.

[21] Sabin F. R. (1917). Preliminary note on the differentiation of angioblasts and the method by which they produce blood-vessels, blood-plasma and red blood-cells as seen in the living chick. *Anatomical Record*.

[22] Palis J., McGrath K. E., Kingsley P. D. (1995). Initiation of hematopoiesis and vasculogenesis in murine yolk sac explants. *Blood*.

[23] Ferkowicz M. J., Starr M., Xie X. (2003). CD41 expression defines the onset of primitive and definitive hematopoiesis in the murine embryo. *Development*.

[24] McGrath K. E., Koniski A. D., Malik J., Palis J. (2003). Circulation is established in a stepwise pattern in the mammalian embryo. *Blood*.

[25] Keller G., Chicha L., Ditadi A. (2012). Primitive erythropoiesis is regulated by miR-126 via nonhematopoietic Vcam-1+ cells. *Developmental Cell*.

[26] Fraser S. T., Isern J., Baron M. H. (2007). Maturation and enucleation of primitive erythroblasts during mouse embryogenesis is accompanied by changes in cell-surface antigen expression. *Blood*.

[27] McGrath K. E., Frame J. M., Fromm G. J. (2011). A transient definitive erythroid lineage with unique regulation of the *β*-globin locus in the mammalian embryo. *Blood*.

[28] Kingsley P. D., Malik J., Emerson R. L. (2006). ‘Maturational’ globin switching in primary primitive erythroid cells. *Blood*.

[29] Yoder M. C., Hiatt K., Dutt P., Mukherjee P., Bodine D. M., Orlic D. (1997). Characterization of definitive lymphohematopoietic stem cells in the day 9 murine yolk sac. *Immunity*.

[30] Yoder M. C., Hiatt K., Mukherjee P. (1997). In vivo repopulating hematopoietic stem cells are present in the murine yolk sac at day 9.0 postcoitus. *Proceedings of the National Academy of Sciences of the United States of America*.

[31] Fraser S. T., Ogawa M., Yu R. T., Nishikawa S., Yoder M. C., Nishikawa S.-I. (2002). Definitive hematopoietic commitment within the embryonic vascular endothelial-cadherin(+) population. *Experimental Hematology*.

[32] Antas V. I., Brigden K. W., Prudence A. J., Fraser S. T. (2014). Gastrokine-2 is transiently expressed in the endodermal and endothelial cells of the maturing mouse yolk sac. *Gene Expression Patterns*.

[33] Gekas C., Dieterlen-Lièvre F., Orkin S. H., Mikkola H. K. (2005). The placenta is a niche for hematopoietic stem cells. *Developmental Cell*.

[34] Gekas C., Dieterlen-Lièvre F., Orkin S. H., Mikkola H. K. A. (2005). The placenta is a niche for hematopoietic stem cells. *Developmental Cell*.

[35] Ottersbach K., Dzierzak E. (2005). The murine placenta contains hematopoietic stem cells within the vascular labyrinth region. *Developmental Cell*.

[36] Robin C., Bollerot K., Mendes S. (2009). Human placenta is a potent hematopoietic niche containing hematopoietic stem and progenitor cells throughout development. *Cell Stem Cell*.

[37] Ivanovs A., Rybtsov S., Welch L., Anderson R. A., Turner M. L., Medvinsky A. (2011). Highly potent human hematopoietic stem cells first emerge in the intraembryonic aorta-gonad-mesonephros region. *Journal of Experimental Medicine*.

[38] Sasaki T., Mizuochi C., Horio Y., Nakao K., Akashi K., Sugiyama D. (2010). Regulation of hematopoietic cell clusters in the placental niche through SCF/Kit signaling in embryonic mouse. *Development*.

[39] Chhabra A., Lechner A. J., Ueno M. (2012). Trophoblasts regulate the placental hematopoietic niche through PDGF-B signaling. *Developmental Cell*.

[40] van Handel B., Prashad S. L., Hassanzadeh-Kiabi N. (2010). The first trimester human placenta is a site for terminal maturation of primitive erythroid cells. *Blood*.

[41] North T., Gu T.-L., Stacy T. (1999). Cbfa2 is required for the formation of intra-aortic hematopoietic clusters. *Development*.

[42] Yokomizo T., Ng C. E., Osato M., Dzierzak E. (2011). Three-dimensional imaging of whole midgestation murine embryos shows an intravascular localization for all hematopoietic clusters. *Blood*.

[43] Yokomizo T., Dzierzak E. (2010). Three-dimensional cartography of hematopoietic clusters in the vasculature of whole mouse embryos. *Development*.

[44] Taoudi S., Gonneau C., Moore K. (2008). Extensive hematopoietic stem cell generation in the AGM region via maturation of VE-Cadherin +CD45 + pre-definitive HSCs. *Cell Stem Cell*.

[45] Boisset J.-C., van Cappellen W., Andrieu-Soler C., Galjart N., Dzierzak E., Robin C. (2010). In vivo imaging of haematopoietic cells emerging from the mouse aortic endothelium. *Nature*.

[46] Peeters M., Ottersbach K., Bollerot K. (2009). Ventral embryonic tissues and Hedgehog proteins induce early AGM hematopoietic stem cell development. *Development*.

[47] Oostendorp R. A. J., Harvey K. N., Kusadasi N. (2002). Stromal cell lines from mouse aorta-gonads-mesonephros subregions are potent supporters of hematopoietic stem cell activity. *Blood*.

[48] Ohneda O., Fennie C., Zheng Z. (1998). Hematopoietic stem cell maintenance and differentiation are supported by embryonic aorta-gonad-mesonephros region-derived endothelium. *Blood*.

[49] Oostendorp R. A. J., Robin C., Steinhoff C. (2005). Long-term maintenance of hematopoietic stem cells does not require contact with embryo-derived stromal cells in cocultures. *Stem Cells*.

[50] Robin C., Ottersbach K., Durand C. (2006). An unexpected role for IL-3 in the embryonic development of hematopoietic stem cells. *Developmental Cell*.

[51] Marshall C. J., Sinclair J. C., Thrasher A. J., Kinnon C. (2007). Bone morphogenetic protein 4 modulates c-Kit expression and differentiation potential in murine embryonic aorta-gonad-mesonephros haematopoiesis in vitro. *British Journal of Haematology*.

[52] Fitch S. R., Kimber G. M., Wilson N. K. (2012). Signaling from the sympathetic nervous system regulates hematopoietic stem cell emergence during embryogenesis. *Cell Stem Cell*.

[53] Chanda B., Ditadi A., Iscove N. N., Keller G. (2013). XRetinoic acid signaling is essential for embryonic hematopoietic stem cell development. *Cell*.

[54] Adamo L., Naveiras O., Wenzel P. L. (2009). Biomechanical forces promote embryonic haematopoiesis. *Nature*.

[55] North T. E., Goessling W., Peeters M. (2009). Hematopoietic stem cell development is dependent on blood flow. *Cell*.

[56] Sugiyama D., Kulkeaw K., Mizuochi C., Horio Y., Okayama S. (2011). Hepatoblasts comprise a niche for fetal liver erythropoiesis through cytokine production. *Biochemical and Biophysical Research Communications*.

[57] Sugiyama D., Kulkeaw K., Mizuochi C. (2013). TGF-beta-1 up-regulates extra-cellular matrix production in mouse hepatoblasts. *Mechanisms of Development*.

[58] Liang B., Hara T., Wagatsuma K. (2012). Role of hepatocyte-derived IL-7 in maintenance of intrahepatic NKT cells and T cells and development of B cells in fetal liver. *Journal of Immunology*.

[59] Chou S., Lodish H. F. (2010). Fetal liver hepatic progenitors are supportive stromal cells for hematopoietic stem cells. *Proceedings of the National Academy of Sciences of the United States of America*.

[60] Hackney J. A., Charbord P., Brunk B. P., Stoeckert C. J., Lemischka I. R., Moore K. A. (2002). A molecular profile of a hematopoietic stem cell niche. *Proceedings of the National Academy of Sciences of the United States of America*.

[61] Iwasaki H., Arai F., Kubota Y., Dahl M., Suda T. (2010). Endothelial protein C receptor-expressing hematopoietic stem cells reside in the perisinusoidal niche in fetal liver. *Blood*.

[62] Tamplin O. J., Durand E. M., Carr L. A. (2015). Hematopoietic stem cell arrival triggers dynamic remodeling of the perivascular Niche. *Cell*.

[63] Isern J., Fraser S. T., He Z., Baron M. H. (2008). The fetal liver is a niche for maturation of primitive erythroid cells. *Proceedings of the National Academy of Sciences of the United States of America*.

[64] Isern J., Fraser S. T., He Z., Baron M. H. (2010). Developmental niches for embryonic erythroid cells. *Blood Cells, Molecules, and Diseases*.

[65] Kawane K., Fukuyama H., Kondoh G. (2001). Requirement of DNase II for definitive erythropoiesis in the mouse fetal liver. *Science*.

[66] Coşkun S., Chao H., Vasavada H. (2014). Development of the fetal bone marrow niche and regulation of HSC quiescence and homing ability by emerging osteolineage cells. *Cell Reports*.

[67] Balduino A., Hurtado S. P., Frazão P. (2005). Bone marrow subendosteal microenvironment harbours functionally distinct haemosupportive stromal cell populations. *Cell and Tissue Research*.

[68] Nakashima K., Zhou X., Kunkel G. (2002). The novel zinc finger-containing transcription factor Osterix is required for osteoblast differentiation and bone formation. *Cell*.

[69] Zou Y.-R., Kottman A. H., Kuroda M., Taniuchi I., Littman D. R. (1998). Function of the chemokine receptor CXCR4 in heaematopolesis and in cerebellar development. *Nature*.

[70] Ma Q., Jones D., Borghesani P. R. (1998). Impaired B-lymphopoiesis, myelopoiesis, and derailed cerebellar neuron migration in CXCR4- and SDF-1-deficient mice. *Proceedings of the National Academy of Sciences of the United States of America*.

[71] Costello P., Sargent M., Maurice D. (2015). MRTF-SRF signaling is required for seeding of HSC/Ps in bone marrow during development. *Blood*.

[72] Adams G. B., Chabner K. T., Alley I. R. (2006). Stem cell engraftment at the endosteal niche is specified by the calcium-sensing receptor. *Nature*.

[73] Kiel M. J., Iwashita T., Yilmaz Ö. H., Morrison S. J. (2005). Spatial differences in hematopoiesis but not in stem cells indicate a lack of regional patterning in definitive hematopoietic stem cells. *Developmental Biology*.

[74] Sacchetti B., Funari A., Michienzi S. (2007). Self-renewing osteoprogenitors in bone marrow sinusoids can organize a hematopoietic microenvironment. *Cell*.

[75] Jiang Y., Bonig H., Ulyanova T., Chang K., Papayannopoulou T. (2009). On the adaptation of endosteal stem cell niche function in response to stress. *Blood*.

[76] Ellis S. L., Grassinger J., Jones A. (2011). The relationship between bone, hemopoietic stem cells, and vasculature. *Blood*.

[77] Nombela-Arrieta C., Pivarnik G., Winkel B. (2013). Quantitative imaging of haematopoietic stem and progenitor cell localization and hypoxic status in the bone marrow microenvironment. *Nature Cell Biology*.

[78] Taichman R. S., Emerson S. G. (1994). Human osteoblasts support hematopoiesis through the production of granulocyte colony-stimulating factor. *Journal of Experimental Medicine*.

[79] Kiel M. J., Yilmaz Ö. H., Iwashita T., Yilmaz O. H., Terhorst C., Morrison S. J. (2005). SLAM family receptors distinguish hematopoietic stem and progenitor cells and reveal endothelial niches for stem cells. *Cell*.

[80] Celso Lo C., Fleming H. E., Wu J. W., Zhao C. X., Miake-Lye S., Fujisaki J. (2008). Live-animal tracking of individual haematopoietic stem/progenitor cells in their niche. *Nature*.

[81] Calvi L. M., Adams G. B., Weibrecht K. W. (2003). Osteoblastic cells regulate the haematopoietic stem cell niche. *Nature*.

[82] Zhang J., Niu C., Ye L. (2003). Identification of the haematopoietic stem cell niche and control of the niche size. *Nature*.

[83] Visnjic D., Kalajzic Z., Rowe D. W., Katavic V., Lorenzo J., Aguila H. L. (2004). Hematopoiesis is severely altered in mice with an induced osteoblast deficiency. *Blood*.

[84] Lymperi S., Ersek A., Ferraro F., Dazzi F., Horwood N. J. (2011). Inhibition of osteoclast function reduces hematopoietic stem cell numbers in vivo. *Blood*.

[85] Miyamoto K., Yoshida S., Kawasumi M. (2011). Osteoclasts are dispensable for hematopoietic stem cell maintenance and mobilization. *Journal of Experimental Medicine*.

[86] Arai F., Hirao A., Ohmura M. (2004). Tie2/angiopoietin-1 signaling regulates hematopoietic stem cell quiescence in the bone marrow niche. *Cell*.

[87] Thorén L. A., Liuba K., Bryder D. (2008). Kit regulates maintenance of quiescent hematopoietic stem cells. *Journal of Immunology*.

[88] Sugiyama T., Kohara H., Noda M., Nagasawa T. (2006). Maintenance of the hematopoietic stem cell pool by CXCL12-CXCR4 chemokine signaling in bone marrow stromal cell niches. *Immunity*.

[89] Qian H., Buza-Vidas N., Hyland C. D. (2007). Critical role of thrombopoietin in maintaining adult quiescent hematopoietic stem cells. *Cell Stem Cell*.

[90] Nilsson S. K., Johnston H. M., Whitty G. A. (2005). Osteopontin, a key component of the hematopoietic stem cell niche and regulator of primitive hematopoietic progenitor cells. *Blood*.

[91] Kiel M. J., Radice G. L., Morrison S. J. (2007). Lack of evidence that hematopoietic stem cells depend on N-cadherin-mediated adhesion to osteoblasts for their maintenance. *Cell Stem Cell*.

[92] Greenbaum A. M., Revollo L. D., Woloszynek J. R., Civitelli R., Link D. C. (2012). N-cadherin in osteolineage cells is not required for maintenance of hematopoietic stem cells. *Blood*.

[93] Kunisaki Y., Bruns I., Scheiermann C. (2013). Arteriolar niches maintain haematopoietic stem cell quiescence. *Nature*.

[94] Méndez-Ferrer S., Michurina T. V., Ferraro F. (2010). Mesenchymal and haematopoietic stem cells form a unique bone marrow niche. *Nature*.

[95] Méndez-Ferrer S., Lucas D., Battista M., Frenette P. S. (2008). Haematopoietic stem cell release is regulated by circadian oscillations. *Nature*.

[96] Yamazaki S., Ema H., Karlsson G. (2011). Nonmyelinating schwann cells maintain hematopoietic stem cell hibernation in the bone marrow niche. *Cell*.

[97] Katayama Y., Frenette P. S. (2003). Galactocerebrosides are required postnatally for stromal-dependent bone marrow lymphopoiesis. *Immunity*.

[98] Katayama Y., Battista M., Kao W.-M. (2006). Signals from the sympathetic nervous system regulate hematopoietic stem cell egress from bone marrow. *Cell*.

[99] Ding L., Saunders T. L., Enikolopov G., Morrison S. J. (2012). Endothelial and perivascular cells maintain haematopoietic stem cells. *Nature*.

[100] Jeansson M., Gawlik A., Anderson G. (2011). Angiopoietin-1 is essential in mouse vasculature during development and in response to injury. *The Journal of Clinical Investigation*.

[101] Tokoyoda K., Egawa T., Sugiyama T., Choi B.-I., Nagasawa T. (2004). Cellular niches controlling B lymphocyte behavior within bone marrow during development. *Immunity*.

[102] Greenbaum A., Hsu Y.-M. S., Day R. B. (2013). CXCL12 in early mesenchymal progenitors is required for haematopoietic stem-cell maintenance. *Nature*.

[103] Omatsu Y., Sugiyama T., Kohara H. (2010). The essential functions of adipo-osteogenic progenitors as the hematopoietic stem and progenitor cell niche. *Immunity*.

[104] Bruns I., Lucas D., Pinho S. (2014). Megakaryocytes regulate hematopoietic stem cell quiescence through CXCL4 secretion. *Nature Medicine*.

[105] Nakamura-Ishizu A., Takubo K., Fujioka M., Suda T. (2014). Megakaryocytes are essential for HSC quiescence through the production of thrombopoietin. *Biochemical and Biophysical Research Communications*.

[106] Olson T. S., Caselli A., Otsuru S. (2013). Megakaryocytes promote murine osteoblastic HSC niche expansion and stem cell engraftment after radioablative conditioning. *Blood*.

[107] Zhao M., Perry J. M., Marshall H. (2014). Megakaryocytes maintain homeostatic quiescence and promote post-injury regeneration of hematopoietic stem cells. *Nature Medicine*.

[108] Fujisaki J., Wu J., Carlson A. L. (2011). *In vivo* imaging of T_reg_ cells providing immune privilege to the haematopoietic stem-cell niche. *Nature*.

[109] Bessis M. C., Breton-Gorius J. (1962). Iron metabolism in the bone marrow as seen by electron microscopy: a critical review. *Blood*.

[110] Chasis J. A., Mohandas N. (2008). Erythroblastic islands: niches for erythropoiesis. *Blood*.

[111] Mohandas N., Prenant M. (1978). Three-dimensional model of bone marrow. *Blood*.

[112] Kusakabe M., Hasegawa K., Hamada M. (2011). c-Maf plays a crucial role for the definitive erythropoiesis that accompanies erythroblastic island formation in the fetal liver. *Blood*.

[113] Sadahira Y., Yasuda T., Kimoto T. (1991). Regulation of Forssman antigen expression during maturation of mouse stromal macrophages in haematopoietic foci. *Immunology*.

[114] Rhodes M. M., Kopsombut P., Bondurant M. C., Price J. O., Koury M. J. (2008). Adherence to macrophages in erythroblastic islands enhances erythroblast proliferation and increases erythrocyte production by a different mechanism than erythropoietin. *Blood*.

[115] Chow A., Huggins M., Ahmed J. (2013). CD169+ macrophages provide a niche promoting erythropoiesis under homeostasis and stress. *Nature Medicine*.

[116] Ramos P., Casu C., Gardenghi S. (2013). Macrophages support pathological erythropoiesis in polycythemia vera and *β*-thalassemia. *Nature Medicine*.

[117] Matsushima T., Nakashima M., Oshima K. (2001). Receptor binding cancer antigen expressed on SiSo cells, a novel regulator of apoptosis of erythroid progenitor cells. *Blood*.

[118] Zamai L., Secchiero P., Pierpaoli S. (2000). TNF-related apoptosis-inducing ligand (TRAIL) as a negative regulator of normal human erythropoiesis. *Blood*.

[119] Sawada K., Krantz S. B., Dessypris E. N., Koury S. T., Sawyer S. T. (1989). Human colony-forming units-erythroid do not require accessory cells, but do require direct interaction with insulin-like growth factor I and/or insulin for erythroid development. *Journal of Clinical Investigation*.

[120] Yoshida H., Kawane K., Koike M., Mori Y., Uchiyama Y., Nagata S. (2005). Phosphatidylserine-dependent engulfment by macrophages of nuclei from erythroid precursor cells. *Nature*.

[121] Miyanishi M., Tada K., Koike M., Uchiyama Y., Kitamura T., Nagata S. (2007). Identification of Tim4 as a phosphatidylserine receptor. *Nature*.

[122] Porcu S., Manchinu M. F., Marongiu M. F. (2011). Klf1 Affects dnase II-Alpha Expression in the central macrophage of a fetal liver erythroblastic Island: a non-cell-autonomous role in definitive erythropoiesis. *Molecular and Cellular Biology*.

[123] Spike B. T., Dibling B. C., Macleod K. F. (2007). Hypoxic stress underlies defects in erythroblast islands in the Rb-null mouse. *Blood*.

[124] Liu X.-S., Li X.-H., Wang Y. (2007). Disruption of palladin leads to defects in definitive erythropoiesis by interfering with erythroblastic island formation in mouse fetal liver. *Blood*.

[125] Jacobsen K., Osmond D. G. (1990). Microenvironmental organization and stromal cell associations of B lymphocyte precursor cells in mouse bone marrow. *European Journal of Immunology*.

[126] Gomez M. R., Talke Y., Goebel N., Hermann F., Reich B., Mack M. (2010). Basophils support the survival of plasma cells in mice. *Journal of Immunology*.

[127] Chu V. T., Fröhlich A., Steinhauser G. (2011). Eosinophils are required for the maintenance of plasma cells in the bone marrow. *Nature Immunology*.

[128] Winter O., Moser K., Mohr E. (2010). Megakaryocytes constitute a functional component of a plasma cell niche in the bone marrow. *Blood*.

[129] Socolovsky M. (2007). Molecular insights into stress erythropoiesis. *Current Opinion in Hematology*.

[130] Paulson R. F., Shi L., Wu D.-C. (2011). Stress erythropoiesis: new signals and new stress progenitor cells. *Current Opinion in Hematology*.

[131] Lenox L. E., Perry J. M., Paulson R. F. (2005). BMP4 and Madh5 regulate the erythroid response to acute anemia. *Blood*.

[132] Perry J. M., Harandi O. F., Paulson R. F. (2007). BMP4, SCF, and hypoxia cooperatively regulate the expansion of murine stress erythroid progenitors. *Blood*.

[133] Perry J. M., Harandi O. F., Porayette P., Hegde S., Kannan A. K., Paulson R. F. (2009). Maintenance of the BMP4-dependent stress erythropoiesis pathway in the murine spleen requires hedgehog signaling. *Blood*.

[134] Yanai N., Satoh T., Obinata M. (1991). Endothelial cells create a hematopoietic inductive microenvironment preferential to erythropoiesis in the mouse spleen. *Cell Structure and Function*.

[135] Tsuchiyama J., Mori M., Okada S. (1995). Murine spleen stromal cell line SPY3-2 maintains long-term hematopoiesis in vitro. *Blood*.

[136] Justesen J., Stenderup K., Ebbesen E. N., Mosekilde L., Steiniche T., Kassem M. (2001). Adipocyte tissue volume in bone marrow is increased with aging and in patients with osteoporosis. *Biogerontology*.

[137] Sugimura R., Li L. (2010). Shifting in balance between osteogenesis and adipogenesis substantially influences hematopoiesis. *Journal of Molecular Cell Biology*.

[138] Gimble J. M., Robinson C. E., Wu X., Kelly K. A. (1996). The function of adipocytes in the bone marrow stroma: an update. *Bone*.

[139] Naveiras O., Nardi V., Wenzel P. L., Hauschka P. V., Fahey F., Daley G. Q. (2009). Bone-marrow adipocytes as negative regulators of the haematopoietic microenvironment. *Nature*.

[140] DiMascio L., Voermans C., Uqoezwa M. (2007). Identification of adiponectin as a novel hemopoietic stem cell growth factor. *The Journal of Immunology*.

[141] Omatsu Y., Seike M., Sugiyama T., Kume T., Nagasawa T. (2014). Foxc1 is a critical regulator of haematopoietic stem/progenitor cell niche formation. *Nature*.

[142] Torisawa Y.-S., Spina C. S., Mammoto T. (2014). Bone marrow-on-a-chip replicates hematopoietic niche physiology in vitro. *Nature Methods*.

